# Twenty Years of Equine Piroplasmosis Research: Global Distribution, Molecular Diagnosis, and Phylogeny

**DOI:** 10.3390/pathogens9110926

**Published:** 2020-11-08

**Authors:** Sharon Tirosh-Levy, Yuval Gottlieb, Lindsay M. Fry, Donald P. Knowles, Amir Steinman

**Affiliations:** 1 Koret School of Veterinary Medicine, The Hebrew University of Jerusalem, Rehovot 7610001, Israel; gottlieb.yuval@mail.huji.ac.il (Y.G.); amirst@savion.huji.ac.il (A.S.); 2 Department of Veterinary Microbiology and Pathology, Washington State University, Pullman, WA 99164, USA; lfry@wsu.edu (L.M.F.); dknowles@wsu.edu (D.P.K.); 3 Animal Disease Research Unit, Agricultural Research Service, US Department of Agriculture, Pullman, WA 99164, USA

**Keywords:** equine piroplasmosis, *Theileria equi*, Babesia caballi, equine, genotyping

## Abstract

Equine piroplasmosis (EP), caused by the hemoparasites *Theileria equi*, *Theileria haneyi*, and *Babesia caballi*, is an important tick-borne disease of equines that is prevalent in most parts of the world. Infection may affect animal welfare and has economic impacts related to limitations in horse transport between endemic and non-endemic regions, reduced performance of sport horses and treatment costs. Here, we analyzed the epidemiological, serological, and molecular diagnostic data published in the last 20 years, and all DNA sequences submitted to GenBank database, to describe the current global prevalence of these parasites. We demonstrate that EP is endemic in most parts of the world, and that it is spreading into more temperate climates. We emphasize the importance of using DNA sequencing and genotyping to monitor the spread of parasites, and point to the necessity of further studies to improve genotypic characterization of newly recognized parasite species and strains, and their linkage to virulence.

## 1. Current Knowledge of Equine Piroplasmosis

Equine piroplasmosis (EP) is a tick-borne disease of equines caused by the eukaryotic hemoparasites *Theileria equi*, *Theileria haneyi*, and *Babesia caballi* that has a considerable veterinary and economic impacts on the horse industry worldwide [1,2,3,4,5]. The parasites belong to the phylum Apicomplexa and to the order Piroplasmida [6]. EP is considered a reportable disease by the World Organization for Animal Health (OIE) (https://www.oie.int/animal-health-in-the-world/oie-listed-diseases-2020/, 15 April 2020). It is estimated that 90% of the global horse population resides in EP-endemic areas, and therefore many studies have investigated the occurrence, prevalence, risk factors, and characteristics of these parasites in different parts of the world. 

### 1.1. Life Cycle, Vectors, and Transmission

**Figure 1:**
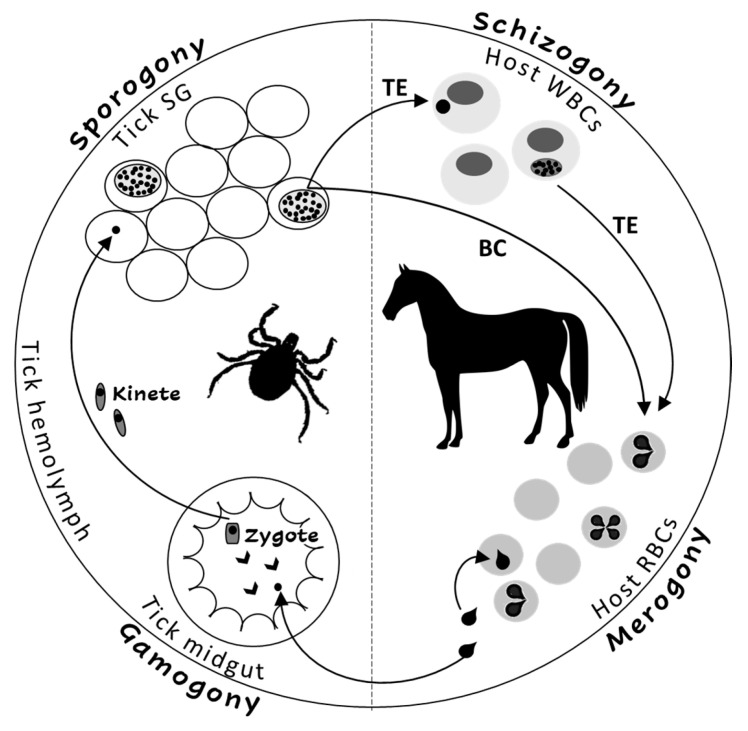
The life cycle of *Theileria equi* (TE) and *Babesia caballi* (BC) in the tick vector and in the equine host. RBC—equine red blood cells, WBC—equine while blood cells, SG—tick salivary glands.

The *Theileria* and *Babesia* genera belong to the families Theileriidae and Babesiidae within the phylum Apicomplexa. The life cycles of both parasites include sexual (gamogony) and asexual (sporogony) replicative stages within the tick vector and asexual replicative stages within the equine host [2,3]. Asexual replication (merogony) in equine erythrocytes is common to both parasites, and *T. equi* (and likely, *T. haneyi*) also undergoes asexual schizogony within equine lymphocytes and monocytes prior to invasion to erythrocytes [7] (Figure 1). The term piroplasmosis derives from the pear-shaped appearance of the intra-erythrocytic stages of these parasites (merozoites). Replication in erythrocytes ultimately leads to cell rupture and the release of merozoites that invade additional cells [2,3,8].

The main route of transmission to equids is by tick feeding. Over 30 species of ticks have been described as vectors of one or both *T. equi* and *B. caballi*, including the genera *Hyalomma*, *Rhipicephalus Dermacentor*, *Amblyomma*, and *Haemaphysalis* [8]. Transstadial transmission was recorded for both parasites in several tick species; however, transovarian transmission was only recorded for *B. caballi* [8]. Therefore, the main reservoir for *T. equi* is in the equine host, whilst for *B. caballi* it is the vector ticks [8]. 

Transplacental transmission in the equine host has been reported for *T. equi* and may lead to abortion, the birth of a sick foal with peracute neonatal EP, or the birth of unapparent carrier foal [9,10,11,12,13,14,15,16,17,18,19]. In some endemic areas, *T. equi* is considered to be a major cause of abortion [20,21]; however, the role of this parasite as a cause of abortion is not well established [22]. Iatrogenic transmission is also possible; there are several reports of infections resulting from blood transfusions, and from sharing of surgical equipment or needles [2,3,5,20]. However, these types of transmission probably do not have a major role in the epidemiology of EP.

### 1.2. Clinical Disease

Clinical disease in EP is mainly attributed to intravascular hemolytic anemia caused by parasite replication and damage to erythrocytes [2,3,20,23]. The clinical signs are similar following infection with both parasite species; however, clinical presentation tends to be more severe in cases of *T. equi* infection [2,3,20]. The incubation period ranges between 12 and 19 days for *T. equi* and between 10 and 30 days for *B. caballi* [2]. Common clinical signs are non-specific and derive from the hemolytic anemia. These include fever, inappetence, icterus, hemoglobinuria, pale mucus membranes (MM), tachycardia, and tachypnea. Thrombocytopenia has also been described. In severe cases, edema and hemorrhage might develop and may eventually lead to organ failure. Gross pathologic findings may include hepatomegaly, splenomegaly, enlarged kidneys, multifocal edema, and hemorrhages [2,3,20,23]. Anecdotal cases of EP-associated hyphema [24], cardiac arrythmias [25], and inflammatory myopathy [26] have also been reported.

Clinical manifestations following infection with either parasite species range from unapparent infection to life threatening disease. Most infected horses remain asymptomatic [2,3,20], while clinically infected horses may develop peracute, acute, subacute, or chronic disease presentations. Peracute disease is life-threatening and has been mostly described in cases of neonatal EP [2,3,20]. Acute disease is characterized by overt presentation of characteristic EP clinical signs, subacute disease manifests milder clinical signs, and chronic disease presents with non-specific signs and mild clinical pathology abnormalities [2,3,20,23,27]. The factors associated with the severity of clinical disease are unknown. Acute disease is more often observed in infections of naïve adult horses, and is less common in equine populations in endemic areas [2,3,20,23]. Stress has been suggested to induce more severe clinical signs, although the evidence to support this assumption is limited [28]. In contrast to *T. equi,* the newly identified *T. haneyi* rarely causes clinical signs, even in splenectomized horses [29].

Regardless of the initial clinical presentation, without treatment, horses infected with EP usually remain persistent subclinical (unapparent) carriers for prolonged periods of time. Carriage of *T. equi* is usually life-long, while *B. caballi* infection may be self-limiting after up to four years [2,3,20].

### 1.3. Immunity, Treatment, and Control

Carriage of parasites usually results in an immune response sufficient to prevent severe disease [2,3]. The precise immune mechanisms involved are not fully elucidated. Both innate and adaptive immunity appear to be necessary for parasite control, and splenectomy leads to severe clinical disease in *T. equi-*infected horses. Antibodies are first detected seven to 11 days after infection, and peak 30 to 54 days after infection [2,3,30,31,32]. 

The most widely used treatment for EP is imidocarb dipropionate [2,3,5]. *Theileria equi* is considered to be more resistant to treatment than *B. caballi*, and requires higher dosages and longer durations of therapy [33,34,35,36]. Two intramuscular (IM) injections 24 h apart of 2 mg/kg are recommended for the treatment of *B. caballi*, and four injections 72 h apart of 4 mg/kg are recommended for *T. equi* [33]. Although this drug is relatively safe, the latter dosage is near its 50% lethal dose (LD_50_), and may cause adverse signs of toxicity or even death (donkeys being more sensitive than horses) [2,3,5,35,36]. Since the administration of 4 mg/kg of imidicarb dipropionate often causes colic in horses, animals are often co-treated with flunixin meglumine or buscopan. Although complete parasite clearance is usually possible, several imidocarb diproprionate treatment cycles may be required [36]. Furthermore, imidocarb diproprionate-resistant parasites have been reported [34]. 

Various other chemotherapeutic agents have been reported to be potentially used against EP, with variable efficacy, mostly in vitro. Among these are anti-malaria compounds [37,38,39], antimicrobial agents [40,41,42,43,44,45], parasite metabolism inhibitors [34,46,47,48,49,50,51,52,53], replication inhibitors [54,55], pyrimidine synthesis inhibitors [56,57], and various plant-derived compounds [58,59,60,61,62,63]. However, most of these options have never been tested in vivo, and none is widely used. 

Since no effective, commercially available vaccines against EP are yet available, control is based on a combination of drug therapy, vector control, and restricted transport of infected horses. The aims of treatment and control strategies differ between endemic and non-endemic regions. In non-endemic areas the aim is to keep the area disease-free. Thus, treatment of infected horses is aimed at complete clearance of infection, while control is mainly based on monitoring and restricting the entrance of infected horses. Several non-endemic countries, including the United States, Australia, and Japan deny entrance of seropositive horses, and either export, quarantine, or euthanize any positive animal within the country [1,2,3,5,20,64,65]. In addition to quarantine, horses transported from endemic to non-endemic areas usually require treatment with acaricides to prevent introduction of vector ticks (https://www.oie.int/index.php?id=169&L=0&htmfile=chapitre_equine_piroplasmosis.htm). In endemic areas, unapparent carriage and the development of premonition are usually encouraged, rather than parasite clearance, to prevent clinical outbreaks. Thus, treatment is usually aimed only to reduce clinical sings of acute infection, while strategic use of acaricides is recommended to reduce, but not eliminate, exposure to ticks [1,2,3,5,20].

### 1.4. Diagnosis

Diagnosis of EP infection is important to identify unapparent carriers, especially prior to transport into non-endemic areas, and to identify EP as a cause of disease clinically ill animals, especially due to the non-specific nature of clinical signs in EP infection. Various diagnostic techniques have been reported based on clinical signs, microscopic examination, culture, serology, and molecular assays [2,3,5,66]. 

Traditionally, identification of piriform parasites in Giemsa-stained blood smears was the diagnostic method of choice in clinical cases [2,3,5,66]. However, the sensitivity of this method is low, leading to false negative results in many chronic and subclinical cases, when parasite loads are low. In vitro culture methods proved more sensitive and specific; however, these methods are time-consuming, require fresh blood samples and skilled personnel, and therefore are not frequently used as routine diagnostic tests [2,3,5,20,66,67,68,69,70,71].

Serological diagnosis has better sensitivity and specificity for the detection of unapparent carrier horses; however, these assays do not provide information on current parasite load for interpretation of clinical disease states. Several EP-specific serologic assays, comprised of various methods, including a complement fixation test (CFT) [72,73], an indirect immunoflorescent antibody test (IFAT) [72,73], and an enzyme-linked immunosorbent assay (ELISA) [74,75,76,77], have been developed. The CFT is very specific; however, it may give false negative results, especially after treatment and with chronicity, since IgG(T) is not complement-fixing. IFAT is more sensitive than the CFT and remains positive in chronic cases; however, interpretation of the results is subjective and difficult to standardize [2,3,5,20,68,72,73]. Different ELISA tests were developed to detect EP infection, including an indirect ELISA (iELISA) [74] and a competitive ELISA (cELISA) [78]. To improve the standardization and performance of these tests, several cELISA assays were developed using purified recombinant antigens, and are currently the United States Department of Agriculture (USDA) and OIE recommended tests for international horse transport screening. The use of a single epitope also reduces the chance of cross-reactivity between the parasites. The immunodominant *T. equi* surface antigens equine merozoite antigen (*ema*)*-1* and *ema-2*, and the *B. caballi* rhoptry-associated protein (*rap*)*-1* were successfully used and proven superior to IFAT and CFT in several studies [2,3,5,20,30,68,74,76,77,79,80,81]. Nevertheless, some heterogeneity has been recorded between isolates, and the USDA-approved *B. caballi rap-1* cELISA assay did not detect infected horses in South-Africa and in the Middle East [82,83,84].

Molecular diagnosis, based on the detection of parasite DNA in equine blood by polymerase chain reaction (PCR), is gaining popularity for the detection of parasites in both clinical and carrier animals. These methods are more sensitive than microscopic examination, and more clinically useful than serology, since they represent current infection. These methods can also be designed to distinguish between parasite species or genotypes. Currently, these methods are more often used for research than in clinical practice [2,3,5,20,68,85,86,87,88,89,90,91,92]. Numerous assays targeting one or multiple EP parasites, including conventional PCR [86,87], nested PCR (nPCR) [90,93], real-time PCR (rtPCR) [82,94,95,96,97,98,99], multiplex PCR (mPCR) [87,89], reverse line blot (RLB) [100,101], and loop mediated isothermal amplification (LAMP) [85,88,91], have been developed. Several of these assays were determined to have high sensitivity, with a detection limit of 10^−7^% parasitized erythrocytes (PE) [2,3,5,20,68,85,86,87,88,89,90,91,92]. Quantitative methods, such as rtPCR (qPCR), have also been developed, but are mostly applied to increase the sensitivity of parasite detection, and are rarely used to evaluate parasite loads [94,95,96,97,98,99,102]. 

Numerous studies aimed to evaluate the sensitivity and specificity of various serological and molecular tests and to compare between them [72,73,74,75,83,85,86,90,103,104,105,106,107,108,109,110,111,112,113,114,115,116,117,118,119,120]. To date, none of the methods was found to be ultimately superior for the detection of chronically infected horses. Therefore, the use of more than one detection method is recommended for better screening of unapparent carriers [121]. In addition, it has been shown that anti-*T. equi* antibodies can be detected by serological tests up to a year after parasite clearance [122], emphasizing the problematic use of these assays for regulatory purposes.

## 2. Epidemiology

The transmission dynamics of *T. equi* and *B. caballi* are different. In endemic areas, animals are usually exposed at a young age to both parasites and develop premonition. Carriage of *T. equi* is usually life-long; thus, the observed prevalence increases with age and the host is the main reservoir of parasites. The prevalence of *B. caballi*, on the other hand, does not increase with age and is higher in younger animals. Clearance of *B. caballi* is possible, and the parasite is transovarially transmitted by ticks, suggesting the main reservoir of this parasite is the tick [2,3,5,8,20,113,123,124,125,126].

EP is endemic in most parts of the world where competent tick vectors are present. Few countries are considered non-endemic, including the US and Canada, the United Kingdom (UK) and Ireland, Northern Europe, Iceland and Greenland, Singapore, Japan, New Zealand, and Australia. In some of these countries, EP has been reported, but is limited to specific areas and is not widespread or endemic [2,3,5,8,123]. 

The only risk factors consistently associated with EP infection are management practices and tick exposure. Other factors, including host species, breed, age, sex, and activity, have been inconsistently associated with infection (reviewed in: [5,123]). Although most EP-endemic areas are within tropical and temperate regions, recent global warming and increased global transportation have led to the spread of both parasites and vectors to previously non-endemic areas, such as the UK [127]. Some of these areas are suitable habitats for potential vector ticks and are therefore susceptible to epizootic disease spread—hence the significance of OIE monitoring of the distribution and spread of EP, a summary of which is available through the OIE’s new World Animal Health Information Database (WAHIS) (https://www.oie.int/wahis_2/public/wahid.php/Wahidhome/Home, 15 April 2020). 

### A Review of EP Epidemiology in the Last 20 Years

Numerous epidemiological studies were conducted in different locations, on four continents, to assess the prevalence of EP in different parts of the world. Here we review the results of these publications. The PubMed database was searched for publications involving “equine piroplasmosis,” “*Theileria equi*,” or “*Babesia caballi"* in the last 20 years (1 January 2000–1 January 2020). The search resulted in 345 publications, which were subsequently screened for epidemiological studies that utilized serological or/and molecular detection methods (surveys based solely on blood smear analysis were excluded), and involved horses (publications focusing solely on donkeys, zebras, non-equine species, or ticks were excluded). A total of 106 studies from 48 countries or regions were included in the analysis. The prevalence of each parasite reported in each paper was summarized according to study location in Table 1. 

**Figure 2:**
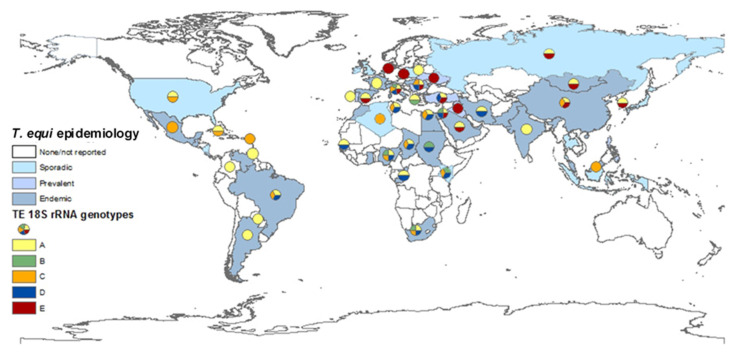
Global prevalence of *T. equi*, and the distribution of *T. equi 18S rRNA* genotypes. The map was constructed based on epidemiological data published in the last 20 years (2000–2019). Endemic: over 30%, prevalent: 10–29%, sporadic: under 10% or singular outbreaks. Genotyping was performed on all sequences submitted to GenBank and classification was based on previously reported clades.

**Figure 3:**
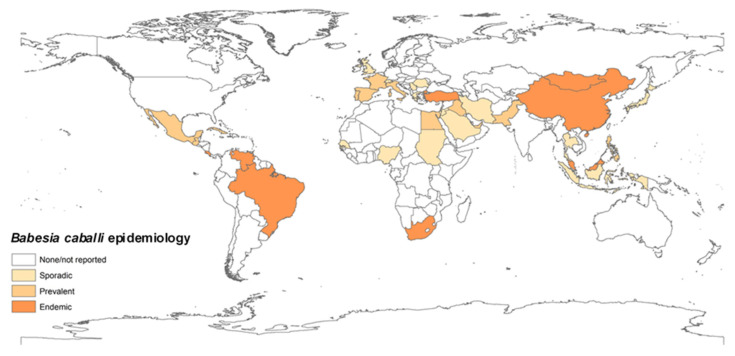
Global prevalence of *B. caballi*. The map was constructed based on epidemiological data published in the last 20 years (2000–2019). Endemic: over 30%, prevalent: 10–29%, sporadic: under 10% or singular outbreaks.

The prevalence of equine piroplasmosis in each country was estimated according to all relevant reports using the following scale. Endemic: over 30%; prevalent: 10–29%; sporadic: under 10% or singular outbreaks. A map representing the global *T. equi* distribution and prevalence was constructed using ArcMap (Esri, Arc GIS desktop 10.6.1.9270) (Figure 2 and Figure 3). 

The global prevalence of equine piroplasmosis and its prevalence in each continent were calculated using a weighted average of the reported prevalence determined by relevant papers for each region. The analysis was performed separately for each parasite, and seroprevalence studies were separated from PCR-based molecular studies (Table 2).

*Theileria equi* seroprevalence was evaluated in 67 studies and 39 regions (a total of 72 studies and regions). The reported seroprevalence ranged from 0.9% (2/224) in Korea [163] to 100% in Brazil (*n* = 170) [75]. *Theileria equi* prevalence was evaluated using molecular techniques in 62 studies and 39 regions (a total of 70 studies and regions). The reported prevalence ranged from 0% in Jordan [115] to 96.8% in Nicaragua [170]. In 25 reports from 18 locations, both molecular prevalence and serological prevalence were evaluated with the same cohort. In the majority of cases (20 of 25 reports), the estimated seroprevalence was higher than the molecular prevalence (Table 1). However, the worldwide seroprevalence calculated from all studies was 33.2% (95% CI: 32.69–33.65), while the molecular prevalence was calculated as 34.6% (95% CI: 34.48–34.76) (Table 2).

*Babesia caballi* seroprevalence was evaluated in 55 studies and 31 regions (a total of 56 studies and regions). The reported seroprevalence ranged from 0% in Italy (*n* = 177), Jordan (*n* = 253), South Korea (*n* = 184), the Netherlands (*n* = 300), and Turkey (*n* = 220) [111,114,115,121,193] to 89.4% in Brazil (42/47) [129]. *Babesia caballi* prevalence was evaluated using molecular techniques in 49 studies and 29 regions (a total of 51 studies and regions). The reported prevalence ranged from 0% in Greece, Iran, Italy, Jordan, Mongolia, the Netherlands, South Africa, Sudan, Thailand, Turkey, and the United Kingdom [92,105,111,115,116,118,121,126,148,155,159,168,178,185,194] to 78% in South Africa [94]. In 20 reports from 14 locations, both molecular prevalence and serological prevalence were evaluated for the same cohort. In the majority of cases (16 of 20 reports), the estimated seroprevalence was higher than the molecular prevalence (Table 1). The worldwide seroprevalence was calculated as 20.5% (95% CI: 19.98–20.93), and the molecular prevalence was calculated as 7.5% (95% CI: 6.99–7.95) (Table 2).

Co-infection or co-exposure to both parasites was evaluated in 40 studies and 22 regions (a total of 41 reports and regions). Co-infection prevalence ranged from 0% in Italy (*n* = 294), Japan (*n* = 2019), and Jordan (*n* = 228) [116,161,162] to 74.5% in Brazil (35/47) [129]. 

The overall prevalence of *T. equi* was higher than that of *B. caballi* worldwide and on every continent. The prevalence of both parasites was the highest in Africa, followed by South and Central America, Europe, and Asia (Table 2, all *p* < 0.001). The prevalence in the Mediterranean region and the Middle East was generally higher than that of northern Europe and the Far East (Table 1).

The global seroprevalence of *T. equi* was calculated as 33%, and its molecular prevalence as 35%. The similarity between the serological and molecular prevalence is consistent with its life-long carriage. Seroprevalence was considerably higher in Africa and Latin America (68% and 58% respectively) than in Asia and Europe (27% and 28% respectively), and this difference was milder when assessing the molecular prevalence (Table 2). The global seroprevalence of *B. caballi*, on the other hand, is considerably higher than its molecular prevalence (20% versus 8%) (Table 2). This may be attributed to parasite clearance, with persistence of antibodies, and may also result from the inherent difficulty of detecting parasite DNA when parasitemia is low. The prevalence of *B. caballi* was considerably higher in Latin America, followed by Asia, Africa, and Europe (Table 2). The highest prevalence of both parasites in Latin America likely reflects studies from Brazil, which comprise the bulk of the data from this area (Table 1). Our screening showed that EP is endemic in most parts of the world, and that it is increasingly reported in areas outside of tropical to temperate climates, which were previously considered EP-free. For example, despite frigid winter temperatures, both clinical and subclinical cases of EP have been reported in Belgium [197], Ireland [198], the Netherlands [111], Switzerland [186], and the UK [126]. These trends are likely related to climate change and resultant habitat alteration for known tick vector species [8,199,200,201,202,203], and to the difficulty in identifying unapparent carriers prior to transport to non-endemic areas. The “sporadic” prevalence noted in some areas may be an artifact of limited monitoring, testing, and reporting, and the prevalence of EP in these areas may increase steadily in coming years, especially once tick vectors are established in the areas [1,8]. 

In general, clinical manifestation of EP is less common in endemic areas, since early exposure is likely to induce protection [2,3,20,23]. However, clinical cases have been reported in resident horses in both non-endemic (Poland, The Netherlands, USA) [204,205,206,207] and in endemic areas (Israel, Italy, Romania, Spain) [25,107,208,209,210]. This highlights the fact that in areas which are considered endemic, there are sub-populations of horses that differ in their exposure to vector ticks, and subsequently to infection with EP and to the development of premonition. These sub-populations should be approached differently in application of preventive measures and treatment to reduce the chance of clinical disease [1,2,3,5,20,125].

Both parasites are endemic in similar areas, although *T. equi* is more frequently reported and is usually more prevalent than *B. caballi*. This may reflect the different transmission cycles of these parasites, since the main reservoirs of *B. caballi* and *T. equi* are in ticks and horses, respectively [2,3,8,20,211]. In addition, parasite loads in both clinically affected horses and in unapparent carriers are usually higher in cases of *T. equi* infection than in *B. caballi* infection, increasing the odds it will be detected by a diagnostic test [2,3,209].

In addition to horses, both parasites have been reported in other equids including domestic donkeys [109,137,138,181,212,213,214,215,216,217,218,219], wild donkeys [214,220], mules [109,138,181,212,216], and zebras [102,214,220,221,222,223,224,225]; and in non-equids, including dogs [145,226,227,228,229,230], camels [231,232], cattle [233], and a tapir [234] (recently reviewed in: [5,214]). Donkeys are considered more resistant to infection than horses [217]; however, this assumption is not well established, since the data regarding domestic equids (donkeys and mules) is less comprehensive than in horses, and many surveys use a population of different equine species. Reports in other animals are anecdotal, and the role of other species as a reservoir of EP has not been demonstrated. All reports describing EP in other animals originate from endemic areas of EP in horses.

## 3. Genotyping

Since their discovery in 1901 [235], the taxonomy of these parasites has been challenged, and they have been re-named and re-classified several times. Currently, *B. caballi* is considred a “true *Babesia*,” while *T. equi* (formerly: *B. equi*) has been classified as *Theileria* based on its extra-erythrocytic life stage in lymphocytes and the absence of transovarian transmission in ticks [211]. Molecular investigations indicate that it possesses characteristics of both *Babesia* and *Theileria,* and is possibly placed between the two [6,236,237,238,239]. The full genome of *T. equi* was constructed from an American strain, which had been used in numerous phylogenetic studies evaluating the genetic diversity between and within piroplasm species [238]. Considerable genetic variation has been found within *T. equi*, and recent discoveries of novel, closely related, species, including *T. haneyi*, suggest that current classification of *T. equi* may include several distinct organisms [4,140]. 

Phylogenetic studies investigating inter-species diversity between piroplasms have mainly focused on the *18S rRNA* [6,140,179,236,239,240]*, β-tubulin* [241], mitochondrial genes [237], and *ema-1* genes [242,243], while intra-species diversity and genotyping mainly focused on the *18S rRNA* [17,83,100,102,107,112,140,144,209,244,245]*, T. equi ema-1* [95,107,112,130,209,245], and *B. caballi rap-1* [82,83,84,107,112,130,209] genes. Five *T. equi 18S rRNA* genotypes (A–E) have been identified up to date [89,100,102,163,246,247], and three (A–C) *ema-1* genotypes [95]. Three *B. caballi 18S rRNA* genotypes have been described (A, B1, and B2) [94,100,209], along with three *rap-1* genotypes (A1, A2, B) [82,83,84]. Recently, the identification of *T. equi* by its *18S rRNA* gene has been scrutinized following the discovery of several new species that were indistinguishable from *T. equi* based on this locus, suggesting this classification may actually represent several distinct species [4,140]. No specific genotype had been found to be linked to increased virulence; however, two unrelated studies reported a higher frequency of clinically affected horses are infected with *T. equi 18S rRNA* genotype A [107,209].

Here, we used the sequences deposited in GenBank in order to classify all isolates into genotypes and to evaluate the global distribution of each genotype (Table S1). Although the genome of *T. equi* has been fully sequenced [238], most molecular studies focus on a very limited selection of targets. Most PCR-based methods have been designed to detect either the *18S rRNA* or *ema-1* genes [6,87,88,90,91,92,94,98,179,242]. Few other studies targeted the *ema-2, β-tubulin* or other rRNA and mitochondrial genes [140,237,241,242,243]; however, there are no sufficient data regarding the sequence heterogeneity of these genes isolated from strains in different areas in the world. In the last two decades, the most frequently used gene for *T. equi* genotyping has been the *18S rRNA* gene. Despite concerns raised that this target may not allow clear distinction between closely related *Theileria* species [4,140], the analysis of its various sequences in the GenBank database provided the best basis to determine *T. equi* genotype distribution. 

Since some inconsistencies exist between reports, we chose to re-classify all sequences submitted to GenBank rather than rely on previous reports. In addition, since some of the sequences were only used for species identification, our analysis included many sequences that have not been previously classified, thus expending the database of genotypes isolated in different locations. We chose to use a five-genotype classification system, although several studies have suggested that *T. equi 18S rRNA* should be categorized into three genotypes, such that genotypes B and E, and genotypes C and D may represent variants within one phylogenetic clade [136,248,249]. The distance matrix does show similarity between each pair; however, in our opinion, the phylogenetic distance was sufficient to regard them as different groups.

### 3.1. Theileria equi 18S rRNA Genotypes and Their Global Distribution

The NCBI nucleotide database was screened for “*T. equi”* or “*T. sp.”* and *“18S rRNA*” or “small ribosomal subunit” sequences ranging between 400 and 2000 nucleotides in length (22 September 2019). After screening, a total of 360 *T. equi* sequences, six *T. haneyi* sequences, and seven *T. sp.* isolated from waterbuck were aligned using the MUSCLE function [250] in MEGA7 software [251] (version 7.0.18), along with 10 other *Babesia* and *Theileria* species. The aligned sequences were arranged in groups according to their reading frame, and sequences known to represent all five monophyletic clades were included in each group. A phylogenetic tree was constructed for each group of sequences using neighbor-joining (NJ) algorithm with Tamura-Nei model [252] and gamma distribution (+G) in MEGA7. All sequences were categorized into one of the five previously described clades (A–E) and arranged according to the country origin of isolation. A map representing the geographical distribution of the different *T. equi 18S rRNA* genotypes was constructed using ArcMap (Esri, Arc GIS desktop 10.6.1.9270) (Figure 2).

Of the 360 sequences included in the analysis, 148 were classified as genotype A, 13 as genotype B, 76 as genotype C, 62 as genotype D, and 61 as genotype E (Table S2). The species of animals and the geographical origin of each genotype are specified in Table 3. The majority of sequences were classified as genotype A, which have been submitted from various locations and from all continents (excluding Australia, which is considered EP-free). Genotype B was only reported in Africa and the Mediterranean area. Genotype C was also reported on all continents, but mostly from Brazil. Genotype D was mostly reported in Africa, the Mediterranean region, and the Middle East. Genotype E was only reported in Asia and Europe. The geographical distribution of *T. equi 18SrRNA* genotypes is indicated in Figure 2. 

**Figure 4:**
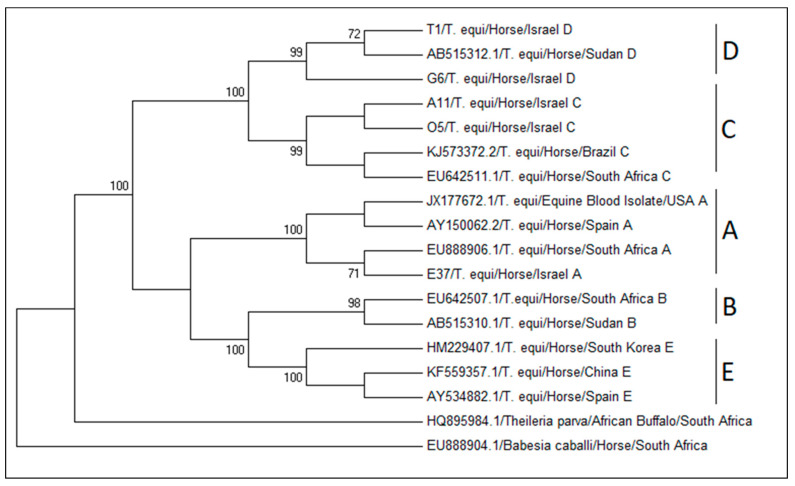
A representative phylogenetic tree of *T. equi 18S* rRNA genotypes. The tree included 18 sequences and 1373 positions. The tree was constructed using maximum likelihood, Tamura-Nei+G+I model with 1000 bootstrap repeats in MEGA7.

Screening for sequences over 1000 nucleotides in length retrieved 195 sequences. These sequences were aligned using MUSCLE function and trimmed to achieve a maximum comparable sequence. The comparable alignment included 1479 positions of 132 sequences. Sequences were grouped according to their clade (A–E) and analyzed to determine conserved and variable domains, and a distance matrix was created to estimate the divergence between sequences and groups as the number of base substitutions per site using Tamura 3-parameter model [253] and gamma distribution (+G) in MEGA7 (Table S3). The divergence between these sequences was less than 0.008 base substitutions per site within each genotype, and between 0.016 and 0.044 base substitutions per site between genotypes (Table 4). Genotypes C and D and genotypes B and E had less evolutionary divergence than that between other genotypes (both 0.016 base substitutions per site) (Table 4). A representative phylogenetic tree appears in Figure 4.

The most widely distributed *T. equi 18S rRNA* genotype is genotype A. This genotype has been isolated in most countries and on all continents. This genotype is also the only one that has been fully sequenced. Although there is no concrete evidence linking any specific genotype to parasite virulence, at least two studies suggest infection with genotype A leads to more severe clinical disease [107,209], and this correlation has also been described during several outbreaks [244]. Moreover, this is the main genotype isolated from ticks (Table 3, [254]) and the only genotype isolated from dogs (Table 3). Genotype C is also widely distributed, and was also found on all continents, except Antarctica. In the Americas, genotypes A and C are the predominant genotypes, with only three Brazilian isolates classified as genotype D (from two horses and one tapir). Aside from these three isolates, genotype D was mainly found in Africa, the Mediterranean region, and the Middle East, and had not been isolated from Northern Europe, the Far East, North and Central America, or the Caribbean region. Genotype E, on the other hand, is mainly found in the Far East, Northern and Eastern Europe and the Middle East, but not in Africa, America or the Caribbean. Genotype B was classified in the fewest number of sequences (*n* = 13) and was only detected in Africa and the Mediterranean region, which supports the separation between genotypes B and E, despite their relatively close phylogenetic distance. The differences in the distribution of each genotype is important in understanding the spread of parasites and the infection dynamics within and between equine populations. Recent studies showed that in endemic areas, many horses are co-infected with several genotypes of *T. equi*, and that the predominant genotype or genotypes differ between equine hosts and subpopulations [102,125,221,255]. Co-infection is also possible with other related species, including *T. haneyi* [28] and *B. caballi* (Table 1). The significance of this co-infection and the relations between parasites or genotypes within the host should be further investigated, since it is likely to be a part of maintaining the enzootic stability in endemic areas. 

### 3.2. Theileria equi ema Genotypes and Their Global Distribution

**Figure 5:**
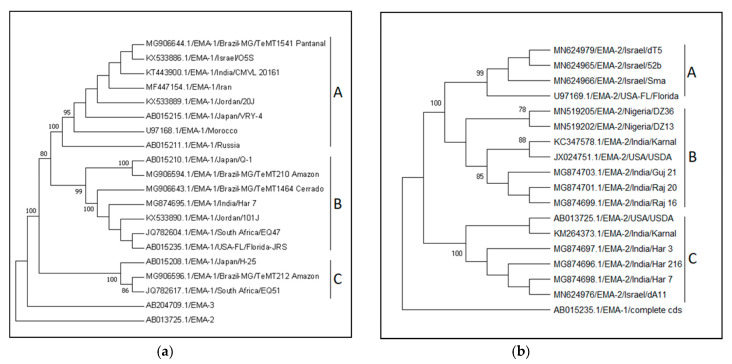
Representative phylogenetic trees of *T. equi ema-1* (**a**) and *ema-2* (**b**) genotypes. (**a**) The tree included 20 sequences and 540 positions. (**b**) The tree included 18 sequences and 800 positions. Both trees were constructed using maximum likelihood, Kimura 2-parameter model with invariable sites (+I) and 1000 bootstrap replicates in MEGA7.

The NCBI nucleotide database was screened for “*T. equi”* and “*ema*” sequences ranging from 500 to 2000 nucleotides in length (29 September 2019). After screening for *ema-1* DNA sequences (and removal of mRNA sequences), 129 sequences were aligned and trimmed as described above. The constructed maximum likelihood phylogenetic tree included 536 positions of 121 *ema-1* sequences, along with one *ema-2* and one *ema-3* sequences using Kimura 2-parameter model [256] with gamma distribution (+G) and 1000 bootstrap replicates in MEGA7 (Figure 5a). Sequences were classified into genotypes according to the constructed tree, and named according to previously described monophyletic clades [95]. A distance matrix was created, as specified above, using the Kimura 2-parameter model [256] and gamma distribution (+G) (Table 5a, Table S4). Comprehensive analysis of all *ema-2* sequences from the gene bank has been previously describes by the authors [209] (Figure 5b, Table 5b and Table S4).

The analysis of *T. equi ema-1* and *ema-2* genes was less informative, since these genes are more conserved, and fewer sequences were available in GenBank. The choice of these loci as targets for molecular investigation was based on the immunodominant properties of the corresponding proteins. Both EMA-1 and EMA-2 proteins have been used as antigens in serologic tests [30,76,79,95,257], and *ema-1* has been used as a target for molecular screening [87,95]. For these purposes, the conserved nature of these genes and antigens was advantageous, in order to develop robust diagnostic techniques. However, it is less informative for genotyping. Combining the sequences of several loci from each isolate may increase the accuracy of the phylogenetic analysis; however, this information was unavailable for most isolates. 

Of the 121 *T. equi ema-1* sequences included in the analysis, 83 were characterized as genotype A, two as genotype B, 23 as genotype C1 and 13 as genotype C2 (Table S4). Genotype A samples were submitted from Brazil, India, Iran, Israel, Japan, Jordan, Morocco, Russia, and Thailand. Genotype B sequences were submitted from Brazil and Japan. Genotype C1 was characterized in Brazil, India, Jordan, South Africa, and the US. Genotype C2 was characterized in Brazil, Japan, and South Africa. A representative phylogenetic tree is illustrated in Figure 5a. The evolutionary divergence within each genotype was lower than 0.014 base substitutions per site, and between genotypes it ranged between 0.02 and 0.155 base substitutions per site (Table 5a). The clade names were assigned according to previous characterization; however, in this analysis, genotypes B and C1 cluster together while genotype C2 is more distant, as confirmed by the distance matrix (Table 5a).

Only 29 *T. equi ema-2* sequences have been submitted to GenBank, of which, 11 were classified as genotype A, 12 as genotype B and six as genotype C (Table S5). Genotype A sequences originated in Israel and the US; genotype B in India, Nigeria, and the US; and genotype C in India, Israel, and the US. The evolutionary divergence within each genotype was lower than 0.004 base substitutions per site, and between genotypes it ranged between 0.011 and 0.059 base substitutions per site [209] (Table 5b).

### 3.3. Babesia caballi Genotypes and Their Global Distribution

**Figure 6:**
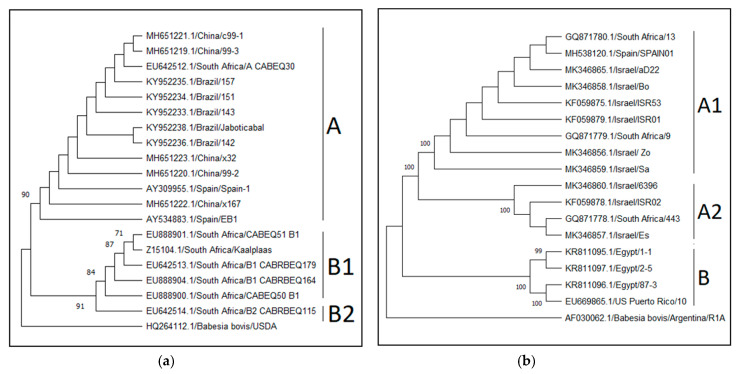
Representative phylogenetic trees of *B. caballi 18S rRNA* (**a**) and *rap-1* (**b**) genotypes. (**a**) The tree included 20 sequences and 1364 positions. The tree was constructed using maximum likelihood, Tamura-Nei model with gamma distribution (+G). (**b**) The tree included 18 sequences and 793 positions. The tree was constructed using maximum likelihood, Kimura 2-parameter model with evolutionarily invariable sites (+I). Both trees were created using 1000 bootstrap replicates in MEGA7.

The NCBI nucleotide database was screened for “*B. caballi”* and “18S” sequences over 400 nucleotides in length (31 September 2019). After screening and removal of unrelated sequences, 133 sequences were aligned using the MUSCLE function [250] in MEGA7. The alignment included 19 long sequences, allowing a comparison of 1367 sites. A phylogenetic tree was created using these sequences along with the sequence of the *B. bovis 18S rRNA* gene (Figure 6a). The tree was constructed using maximum likelihood algorithm, Tamura-Nei model with gamma distribution (+G), and 1000 bootstrap replicates in MEGA7. Sequences were classified according to previously described genotypes [100]. 

The remaining sequences were of different segments of the gene and were aligned in two batches. After trimming and removal of two sequences that did not have sufficient range of alignment with either group, the remaining sequences were trimmed twice: The first batch included 57 sequences and 432 nucleotide positions, and the second included 93 sequences and 435 nucleotide positions. A maximum likelihood phylogenetic tree was constructed from each batch, along with the *B. bovis* sequence using Kimura 2-parameter model [256] with gamma distribution (+G) and 1000 bootstrap replicates in MEGA7. Sequences from the first batch were classified into genotypes according to the longer-sequence construct tree, and named according to previously described monophyletic clades [100] (Table S6). One sequence was removed from the analysis as it was distinctly different from the other sequences. Sequences were grouped according to their assigned clade and analyzed to determine the evolutionary divergence between sequences and groups using a distance matrix and Kimura 2-parameter model [256] and gamma distribution (+G) in MEGA7 (Table 6a). Sequences from the second batch could not be analyzed due to insufficient divergence to discriminate between B1 and B2 genotypes.

The NCBI nucleotide database was then screened for “*B. caballi”* and “*rap-1*” or “BC48” sequences over 400 nucleotides in length (31 September 2019). After screening for *rap-1* DNA, 118 sequences were aligned and trimmed as specified above, and two significantly divergent sequences were removed. The remaining sequences were trimmed twice: Once to create a longer segment of 769 nucleotides which included 23 sequences (Figure 6b), and once to create a shorter comparable segment of 327 nucleotides that included 114 sequences. A maximum likelihood phylogenetic tree was constructed from these sequences, along with *rap-1* orthologues from three other *Babesia* spp. The analysis of the longer sequence was preformed using Kimura 2-parameter model (Kimura, 1980) with regard to evolution invariable sites (+I), while the shorter segment was analyzed using Kimura 2-parameter model [256] with gamma distribution (+G) and regard to evolution invariable sites (+I). Both analyses included 1000 bootstrap replicates and were conducted in MEGA7. Sequences were classified into genotypes according to the longer-sequence construct tree, and named according to previously described monophyletic clades [84]. Sequences were grouped according to their assigned clade and a distance matrix was created using Kimura 2-parameter model [256] and gamma distribution (+G) in MEGA7 (Table 6b, Table S7).

Less information is available regarding *B. caballi* genotyping. Three *18S rRNA* and three *rap-1* genotypes have been identified [82,83,84,100,136], but due to the paucity of sequences submitted to GenBank, little can be deduced about the distribution of the different genotypes in different parts of the world. Although the *rap-1* gene and protein are considered fairly conserved, the serological assay based on this protein failed to detect infection in some cases [82,83,84], which may be related to the difference between the A and B genotypes. Recent work demonstrated a correlation between *B. caballi 18S rRNA* and *rap-1* genotypes [209], making the classification more robust. Additional molecular data from various locations should be gathered and analyzed in order to understand the global epidemiology of this parasite.

A total of 56 *B. caballi 18S rRNA* sequences were included in the analysis, of which 27 were classified as genotype A, 15 as genotype B1 and 14 as genotype B2 [82,84] (Table S6). Genotype A sequences originated from Brazil, China, Croatia, Italy, Mongolia, Saint Kitts and Nevis, South Africa, and Spain. The B1 sequences were from China, Italy, Mongolia, Senegal, and South Africa, and the genotype B2 sequences were from Ethiopia, Guinea, Italy, Mongolia, and South Africa. Most of the sequences were from infected horses; however, eight of the sequences were from ticks (in China, Ethiopia, Guinea, and Italy, four of genotype B1 and four of B2), one was from a donkey in Italy (genotype A), and one was from dog in Croatia (genotype A). Analysis of the evolutionary divergence between these 56 sequences revealed less than 0.017 base substitutions per site within each genotype, and between 0.005 and 0.065 base substitutions per site between genotypes (Table 6a).

A total of 112 *B. caballi rap-1* sequences were included in the analysis, of which 15 were classified as genotype A1, four as genotype A2, and 93 as genotype B [84] (Table S6). Genotype A1 sequences originated from Israel, South Africa, and Spain, A2 sequences were from Israel and South Africa, and genotype B sequences were from Brazil, China, Cuba, Egypt, Indonesia, Mongolia, Thailand, and the USA (Puerto Rico). Analysis of the evolutionary divergence between the 112 sequences revealed less than 0.021 base substitutions per site within each genotype, and between 0.121 and 0.320 base substitutions per site between genotypes (Table 6b). When the longer sequences were evaluated, genotype B was divided into two sub-genotypes (Figure 6); however, when the shorter sequences were compared, the divergence between these two sub-genotype was low (0.008 base substitutions per site) and more data (a longer sequence) are required to correctly classify many of the sequences in this group. 

## 4. Concluding Remarks

The current understanding of the global prevalence of EP is based on prevalence studies from different areas and on annual reports of clinical cases or pre-export test results to the OIE. These sources differ in size and in diagnostic methods, and therefore any comparison should be made with caution. In this review, we attempted to combine the serologic and molecular data from the last 20 years in order to provide a comprehensive view of the epidemiology of EP worldwide.

The analysis of data from all publications provides more robust information than any individual survey. Moreover, analysis of scientific publications provides more detailed information regarding the spread of disease than the data available in the OIE database. Official reports are often lacking and there are discrepancies between OIE data and scientific publication data, especially in endemic areas where clinical cases are less common. For example, EP was not reported from China or Italy in the last 20 years, while numerous studies evaluating EP prevalence and genotypes demonstrate these countries are endemic for both parasites [27,80,107,108,116,117,119,121,141,142,144,145,159,160]. Epidemiological studies also provide information on the prevalence of EP and of each parasite individually, rather than merely the presence of the disease in any country. Table S8 summarizes the OIE reports and list 20 countries in which EP was not reported to the OIE in the last 20 years, but were positive, and even highly endemic in epidemiological studies. 

Evaluating the molecular and serological prevalence based on combined results from various reports should strengthen the statistical power of the analysis. However, studies differ in their selection of the study population, and use various detection methods (within the serological or molecular groups). Since the main factor which affects EP prevalence is tick exposure [5,123], the results of studies that sampled stabled horses or well-groomed sport horses may have different results than studies that sampled pasture-kept horses within the same area. Diagnostic methods differ in their sensitivity and specificity, and interpretation of the results sometimes requires skilled personnel [2,3,5,68]. For this reason, we elected to analyze only the results of serological and molecular studies, and omitted reports based solely upon microscopic examination, which is significantly less sensitive and specific. Nevertheless, comparing or combining the results from different studies should be interpreted with caution. Moreover, the data from different countries varies in its magnitude and quality, since there is much EP research and surveillance in some countries, and very limited to absent data from others. This fact biases the results when assessing regional data, which will be most representative of areas in which data collection is extensive. 

This study provides comprehensive analysis of the current knowledge regarding the prevalence and molecular epidemiology of EP. We concluded that EP is endemic in most parts of the world, and is spreading further into more temperate climate zones previously considered parasite-free. The use of genotyping to monitor the spread of infection is important for better surveillance and control. In the future, whole genome sequencing of the different genotypes should be established to better understand differences in virulence and the clinical impact of different parasite strains. Since different approaches for treatment and control should be implemented in endemic versus non-endemic areas, the fact that most parts of the world harbor these parasites should highlight the importance of distinguishing between susceptible and resistant equine subpopulations in order to reduce the clinical and economic impacts of this disease.

## Figures and Tables

**Table 1:** The prevalence of equine piroplasmosis (EP) in various locations, as was reported in the literature in the last 20 years (1 January 2000–1 January 2020). Only studies which applied serological or/and molecular diagnostic methods were included.

		*T. equi*		*B. caballi*				
Location	*N*	Sero-Prevalence (%)	Prevalence (%)	Sero-Prevalence (%)	Prevalence (%)	Co-Infection (%)	Method *	Ref.
Argentina	180	65					iELISA	[74]
Azores	143	2.8	2.8				cELISA/nPCR	[127]
Balkan	142		22.5		2.1	0.7	mPCR	[128]
Brazil	47	81		90		75	ELISA	[129]
Brazil	35		85.7				qPCR/	[99]
Brazil	487	91	59.7	83	12.5	8.6	IFAT/MRT-PCR	[130]
Brazil	582	21.6		54.1			CFT/cELISA	[131]
Brazil	170	100	63.5				IFAT/nPCR	[75]
Brazil	170	95.9					ELISA	[79]
Brazil	579	81.1					IFAT	[132]
Brazil	314		81				rtPCR	[133]
Brazil	198	78.3		69.2		50	cELISA	[134]
Brazil	400	61					ELISA	[135]
Brazil	39	43.5	38.5	7.7	60	28.2	ELISA/PCR	[136]
Brazil	430	87.4	87.9/90.5	58.6	9.3/7.9	8.8	cELISA/dqPCR/qPCR	[97]
Brazil	528		84.3		23.5		nPCR	[137]
Brazil	359	33.6					iELISA	[138]
Brazil	170	61.8		52.9		49.4	ELISA	[139]
Chad	96		20.8				PCR	[140]
Chad	59		72.8				PCR	[140]
China	70	40		24.3		15.7	ELISA	[141]
China	55		81.8		56.3		LAMP	[85]
China	1990	11.5		51.2		7.6	cELISA	[142]
China	723		40.8				PCR	[143]
China	242		30.2		2.9	2.1	nPCR	[144]
China	56			57.1			ICT	[80]
China	200		39.5		24.5		PCR	[145]
Costa Rica	130	88.5	46.2	69.2	20	62.3/7.7	cELISA/nPCR	[110]
Cuba	100		73		25	20	nPCR	[146]
DR Congo	48		43.7				PCR	[140]
Dubai	105	32.4/33.3		15.3/10.5		12.4	cELISA/IFAT	[104]
Egypt	88	23.9	36.4	17	19.3		IFAT/nPCR	[83]
France	111		80		1.2		PCR	[147]
France	443	58		12.9			CFT	[123]
France	51		29.4				PCR	[140]
France	98		39.8				PCR	[140]
Ghana	30		53.3				qPCR	[99]
Ghana	20		60				PCR	[86]
Greece	544	11		2.2		1.7	cELISA	[148]
Greece	772		44		0		RLB-PCR	[149]
Guatemala	74	92.7	17		16		IFAT/PCR	[150]
Hungary	324	32					cELISA/IFAT	[151]
Hungary	101		49				PCR	[151]
India	5651	32.6					ELISA	[76]
India	426	48.6	19.7				iELISA/nPCR	[109]
Indonesia	235	2.1	0.4	6.4	1.7		cELISA/nPCR	[152]
Iran	100	48	45	2	0	3	IFAT/PCR	[118]
Iran	240		10.8		5.8	1.6	PCR	[153]
Iran	104		22.8				PCR	[154]
Iran	31		96.7		0		PCR	[155]
Iran	126		27.7				PCR	[156]
Israel	216	50.9					ELISA	[157]
Israel	590		26.4				PCR	[158]
Israel	257				9.3		PCR	[84]
Italy	412	12.4		17.9		38.1	IFAT	[117]
Italy	294	8.2	2.7	0.3	0	0	IFAT/PCR	[116]
Italy	300	41	11.7	26	6	14.7	IFAT/PCR	[119]
Italy	1441	31.6		1.2		0.6	IFAT	
Italy	177	41	32.4	0	0		IFAT/PCR	[121]
Italy	160		26.9		0			[159]
Italy	673	39.8		8.9			cELISA	[108]
Italy	135		13.3				PCR	[160]
Japan	2019	2.2		5.4		0	ELISA	[161]
Jordan	253	14.6	0	0	0		cELISA/PCR	[115]
Jordan	288		18.8		7.3	0	mPCR	[162]
Korea	184	1.1		0			cELISA	[114]
Korea	224	0.9					PCR	[163]
Malaysia	306	51.3		63.1		34.3	cELISA	[164]
Mexico	248	45.2		27.4			IFAT	[165]
Mexico	1000		19.7				nPCR	[166]
Mongolia	254	72.8		40.1		30.7	ELISA	[167]
Mongolia	39		25.6		17.9		mPCR	[87]
Mongolia	510	78.8	66.5	65.7	19.1		IFAT/PCR	[113]
Mongolia	250	19.6	6.4	51.6	6.1	10.4/2.5	ELISA/nPCR	[112]
Mongolia	192		92.7		0		nPCR/mPCR	[168]
Mongolia	1282	33		14.2		16.8	ELISA	[169]
Morocco	578	67					cELISA	[106]
Netherlands	300	4	5	0	0		IFAT/RLB-PCR	[111]
Nicaragua	93		96.8		26.8		PCR	[170]
Nigeria	342	73.1		4.4			cELISA	[171]
Pakistan	430	41.2		21.6		10.2	cELISA	[172]
Palestine	108	29.6					ELISA	[157]
Philippines	105	11.4	24.8	10.4	1.9		ICT/PCR	[173]
Poland	76		1.3				PCR	[174]
Portugal	162	17.9		11.1			cELISA	[175]
Portugal	162	9.3	1.9				cELISA/nPCR	[127]
Romania	178		38.8		4.5		mPCR	[176]
Saudi Arabia	141		42				qPCR	[98]
Saudi Arabia	241	10.4		7.5		3	IFAT	[177]
Senegal	127		16.5		0.01		qPCR	[140]
Slovakia	39		0				PCR	[174]
South Africa	37		91.8		45.9		LAMP	[85]
South Africa	99	97.9	9	51.5	0		IFAT/PCR	[178]
South Africa	488		50		3		RLB-PCR	[100]
South Africa	41	83	80	70	78		IFAT/qPCR	[94]
Spain	181		50.3		0.6		RLB-PCR	[179]
Spain	60	40		28.3		20	IFAT	[180]
Spain	135		17		3		PCR	[181]
Spain	428	50.3		11.4		8.4	cELISA	[182]
Spain	3100	44		21			IFAT	[183]
Spain	235	61.7	66	3.8	29.4		cELISA/mnPCR	[90]
Spain	3368	21		5.6		2.5	cELISA	[184]
Sudan	126	63.5		4.4			ELISA	[185]
Sudan	131		25.2		0		PCR	[185]
Sudan	499		35.9		0		PCR	[92]
Switzerland	689	5.9		3		1.5	IFAT	[186]
Thailand	240	5.42/8.75	1.25	2.5/5	0		ELISA/IFAT/PCR	[105]
Trinidad	93	33.3		68.8		19.4	IFAT	[187]
Trinidad	111		24.3		3.6		PCR	[17]
Tunisia	104		12.5		1.9	1.9	RLB-PCR	[188]
Turkey	108	25					IFAT	[189]
Turkey	481	17.7		2.29		1.46	cELISA	[190]
Turkey	84	23.8		38		5.6	IFAT	[191]
Turkey	125	12.8		9.6		4	IFAT	[192]
Turkey	220	56.8		0			cELISA	[193]
Turkey	203		2.96		1.97		qPCR	[120]
Turkey	125		8.8		0		mPCR	[194]
UK	1242	5.9	0.8	4.4	0	2	IFAT/cELISA/CFT/nPCR	[126]
Ukraine	100		29					[174]
Venezuella	360	50.3		70.6		35.6	cELISA	[195]
Venezuella	694	14		23.2		13	cELISA	[196]
Venezuella	136		61.8		4.4	4.4	mPCR	[196]

* Serology: CFT—complement fixation test, IFAT—indirect immunoflorescent antibody test, ELISA—enzyme-linked immunosorbent assay, cELISA—competitive ELISA, iELISA-indirect ELISA, ICT—immunochroma tographic test. Molecular: PCR—polymerase chain reaction, nPCR—nested PCR, mPCR—multiplex PCR, qPCR—quantitative PCR, rtPCR—real time PCR, RLB-PCR—reverse line blot PCR, LAMP—loop-mediated isothermal amplification.

**Table 2:** Global molecular prevalence (based on PCR) and seroprevalence of equine piroplasmosis, as evaluated by weighted average of all reports listed in Table 1.

	TE Seroprevalence			TE Prevalence			BC Seroprevalence			BC Prevalence		
	(%)	*N*	Ref.	(%)	*N*	Ref.	(%)	*N*	Ref.	(%)	*N*	Ref.
**Worldwide**	33.17	37,398	72	34.55	15,849	70	20.45	27,582	56	7.35	11,840	51
Africa	68.21	1274	6	38.02	1867	14	16.52	696	5	5.14	1614	9
Asia	26.79	16,217	27	29.43	5418	23	24.52	9540	22	8.86	3871	19
Europe	27.89	14,497	20	22.26	4917	19	9.42	1368	17	2.48	4227	13
South America	58.21	5410	19	56.92	3647	14	54.05	3478	12	15.98	2128	10

TE—*T. equi*, BC—*B. caballi*, *N*—cumulative number of horses in all studies, Ref—the number of relevant studies.

**Table 3:** *Theileria equi 18S rRNA* classification into genotypes, using all sequences submitted to GenBank in the last 20 years (2000–2019). The total number of sequences is stated for each genotype, along with the origin of the submitter and the stated hosts.

Genotype	Total	Horse	Origin	Donkey	Origin	Zebra	Origin	Tick	Origin	Dog	Origin	Camel	Origin	Cattle	Origin	Tapir	Origin
**A**	148	122	Brazil, Cuba, France, India, Iran, Israel, Jordan, Mongolia, Romania, Saudi Arabia, South Africa, South Korea, Spain, Trinidad and Tobago, Turkey, US	3	Italy	1	Israel	13	Brazil, China, Columbia, France, India, Italy, Portugal, Tunisia	5	Jordan, Paraguai, Spain, Saudi Arabia	4	Jordan				
**B**	13	3	Jordan, South Africa, Sudan	6	Italy	4	South Africa										
**C**	76	74	Brazil, China, Cuba, Israel, Kenya, Malezia, Mexico, Romania, South Africa					1	Italy					1	Algiria		
**D**	62	44	Brazil, Iran, Israel, Jordan, Romania, South Africa, Sudan, Turkey	7	Italy, Kenya	7	Israel, Nigeria, South Africa					3	Iran			1	Brazil
**E**	61	57	China, Hungary, Iran, Iraq, Jordan, Mongolia, Romania, Russia, Saudi Arabia, South Korea, Spain, Switzerland, Turkey, Ukraine					4	China, Mongolia								

**Table 4:** Analysis of the divergence between *T. equi 18S rRNA* sequences over 1000 bases in length submitted to GenBank between 2000 and 2019 (*n* = 195), according to their assigned genotypes. The divergence is displayed as the number of base substitutions per site and was calculated using Tamura 3-parameter model and gamma distribution (+G) in MEGA7.

		Within Genotype	Between Genotypes			
Genotype	*N*		A	B	C	D
**A**	55	0.004				
**B**	5	0.006	0.037			
**C**	40	0.004	0.030	0.038		
**D**	22	0.004	0.031	0.034	0.016	
**E**	10	0.008	0.039	0.016	0.044	0.041

**Table 5:** Analysis of the divergence between *T. equi ema-1* (**a**) and *ema-2* (**b**) sequences submitted to GenBank between 2000 and 2019, according to their assigned genotypes. The divergence is displayed as the number of base substitutions per site and was calculated using Tamura 2-parameter model and gamma distribution (+G) in MEGA7.

(a) *ema-1*		Within Genotype	Between Genotypes		
Genotype	*N*		A	B	C1
**A**	83	0.004			
**B**	2	0.000	0.075		
**C1**	23	0.002	0.081	0.020	
**C2**	13	0.014	0.155	0.125	0.123
**(b) *ema-2***		**Within Genotype**	**Between Genotypes**		
**Genotype**	***N***		**A**	**B**	
**A**	11	0.000			
**B**	12	0.004	0.011		
**C**	6	0.001	0.059	0.055	

**Table 6:** Analysis of the divergence between *B. caballi 18S rRNA* (**a**) and *rap-1* (**b**) sequences submitted to GenBank between 2000 and 2019, according to their assigned genotypes. The divergence is displayed as the number of base substitutions per site and was calculated using Kimura 2-parameter model and gamma distribution (+G) in MEGA7.

(a) *18S rRNA*		Within Genotype	Between Genotypes		
Genotype	*N*		A	B1	
**A**	27	0.005			
**B1**	15	0.01	0.065		
**B2**	14	0.017	0.052	0.031	
**(b) *rap-1***		**Within Genotype**	**Between Genotypes**		
**Genotype**	***N***		**A1**	**A2**	**B1**
**A1**	15	0.001			
**A2**	4	0	0.120		
**B1**	87	0.015	0.310	0.277	
**B2**	6	0.02	0.312	0.278	0.008

## Supplementary Materials

The following are available online at https://www.mdpi.com/2076-0817/9/11/926/s1, Table S1: Workflow of the screening and genotypic characterization of published sequences of *T. equi 18S rRNA* and *ema-1* gene sequences; and *B. caballi 18S rRNA* and *rap-1* gene sequences from the GenBank, Table S2: *Theileria equi 18S rRNA* gene sequences obtained from GenBank and their classification into genotypes, Table S3: Estimates of evolutionary divergence between *T. equi 18S rRNA* sequences, Table S4: Estimates of evolutionary divergence between *T. equi ema-1* gene sequences, Table S5: Estimates of evolutionary divergence between *T. equi ema-2* gene sequences, Table S6: Estimates of evolutionary divergence between *B. caballi 18S rRNA* gene sequences, Table S7: Estimates of evolutionary divergence between *B. caballi rap-1* gene sequences, Table S8: Cases of equine piroplasmosis (EP) reported to the OIE in the last 20 years (2000-2019).
